# Symmetry-guided explainable deep learning for colon cancer diagnosis: model benchmarking, cross-validation, statistical analysis, and explainability via ablation studies

**DOI:** 10.3389/frai.2026.1762636

**Published:** 2026-04-14

**Authors:** Anvaya Solanki, Dewansh Gopani, Monika Mangla, Nonita Sharma, Mu’azu Jibrin Musa, R. S. M. Lakshmi Patibandla

**Affiliations:** 1 Department of Information Technology, Dwarkadas J. Sanghvi College of Engineering, Mumbai, India; 2 Indira Gandhi Delhi Technical University for Women, New Delhi, India; 3 Department of Electronics and Telecommunications Engineering, Ahmadu Bello University, Zaria, Nigeria; 4 Department of CSE, Koneru Lakshmaiah Education Foundation, Guntur, AP, India

**Keywords:** colon cancer, deep learning, explainability, Grad-CAM, LIME, SHAP, symmetry

## Abstract

**Introduction:**

Histopathological tissue reveals natural radial and bilateral symmetry in glandular structures, which becomes progressively disrupted during malignant transformation. Leveraging this observation, this work presents a VGG16-based deep learning model enriched with symmetry-aware interpretation for early detection of Colon Adenocarcinoma. The traditional approaches are not straightforward enough and acts as “black boxes” diminishing their clinical adoption and acceptance in real-world scenario. Current research work uses the most recent breakthroughs in deep learning on medical imaging and integrates Explainable AI strategies such as LIME, SHAP, and Grad-CAM into the model to interpret how cancer-induced symmetry distortions influence model decisions.

**Methods:**

This work is experimented on a balanced dataset of 10,000 histopathological scans, including 5,000 Colon Adenocarcinoma tissue samples and 5,000 Benign Colon Tissue samples. This research aims to shed light on how benign tissues preserve consistent symmetric glandular patterns; while cancerous samples exhibit pronounced asymmetry, irregular boundaries, and disrupted structural repetition. Authors further aim to quantify these differences using lightweight 2D symmetry indices, demonstrating a clear separation between normal and malignant tissues.

**Results and Discussion:**

Current research presents a highly precise model for the diagnosis of colon cancer using a VGG16 CNN that achieves an encouraging test accuracy of 99.85%. The model exhibited very high precision, recall, and F1-scores for both classes, normal and cancer, as demonstrated by the classification report. Among various XAI techniques, Grad-CAM demonstrated speed and scalability making it an appropriate choice for its large-scale deployment in healthcare. SHAP, though computationally costly, offered theoretical robustness and great insight. LIME was handy in local interpretability, especially convenient in debugging individual predictions.

## 1 Introduction

Colon cancer has emerged as a prime cause of mortality across the globe (American Cancer Society, 2025). Despite significant advancement in domain of healthcare—primarily in imaging and diagnostic techniques—current diagnostic methods lack in terms of speed, accuracy, and robustness. The primary reason for this limited performance is that these methods rely heavily on subjective interpretation, which may hinder their ability to effectively capture and highlight subtle patterns present in histopathological images. Unfortunately, delayed or inaccurate diagnosis of the disease may lead to exponential decay of the patient and hence early diagnosis of colon cancer is absolutely essential.

Early diagnosis not only vastly enhances patient survival rates but also reduces the burden on health systems across the globe. Colon Adenocarcinoma, the most prevalent type of colon cancer, usually arises from benign polyps over many years, providing a valuable window for screening and intervention. Nonetheless, traditional diagnostic techniques—largely histopathological analysis by experienced pathologists—may be time-consuming, prone to inter-observer variation, and sometimes fail to detect subtle early-stage lesions. Thus, there is an acute need for machine-learning (ML) based tools that can match human expertise while providing consistent and reproducible results (Dagnaw and El Mouthadi, 2023).

Symmetry is quite significant for computer vision, as many natural and biological structures show predictable symmetric patterns. In medical imaging, particularly histopathology, healthy tissue frequently exhibits pronounced radial, bilateral, and translational symmetry in glandular and cellular configurations. On the contrary, progression of disease from benign to Colon Adenocarcinoma causes breaking down of these symmetric arrangements due to uncontrolled growth, uneven nuclei, stromal infiltration, and loss of glandular integrity. Thus, it is imperative to diagnose such symmetrical disruptions, in order to perform disease diagnosis.

For the same, Deep learning (DL) models can be used to learn these symmetry-related patterns. Also, Explainable AI (XAI) techniques (Volkov and Averkin, 2023) can be leveraged to determine if the model’s attention focuses on regions where symmetry is disrupted, which is a hallmark of colon cancer. This research work aims to build upon traditional DL classification by demonstrating that XAI-highlighted regions strongly correspond to areas where symmetry is disrupted.

Over the last few years, Convolutional Neural Networks (CNNs) have demonstrated phenomenal accuracy in medical image analysis, performing at or near human, and in some cases superhuman, levels on a wide range of tasks—from diabetic retinopathy detection to skin lesion classification (Sharma et al., 2021; Sharma N. et al., 2023). However, despite their high performance, these “black-box” models often fail to inspire confidence among clinicians, who reasonably require transparent and explainable evidence before integrating automated systems into clinical practice (Latha et al., 2024). Without interpretability, even highly accurate models remain limited to research settings rather than becoming trusted components of diagnostic workflows.

The present work addresses the research gap by leveraging a VGG16-based CNN trained on a balanced dataset of 10,000 histopathology images, consisting of 5,000 samples of Colon Adenocarcinoma tissue and benign tissue each (Borkowski et al., 2019). More importantly, authors boost this architecture with state-of-the-art XAI techniques—Grad-CAM for spatial attention visualization, LIME for local linear explanations, and SHAP for quantitative feature attribution. Collectively, these XAI approaches provide pixel-accurate heatmaps and numerical scores that help to identify the causal features underlying each prediction, ensuring that the model provides a meaningful and biologically grounded understanding of cancer detection.

The prime contribution of the research work is to enhance the capability of CNN by integrating XAI components to achieve high classification accuracy while delivering actionable explanations of its decision-making process. In this paper, authors first highlight the challenges associated with applying CNNs to histopathological images and review the current landscape of XAI in medical imaging in section 1. Notable research findings achieved by renowned researchers have been summarized in section 2. Thereafter authors describe the dataset, system architecture, and the integration of XAI interpretability tools along with their comparison in section 3. Experimental results demonstrating the efficiency and effectiveness of proposed system is presented in section 4. It describes how the proposed system support pathologists in colon cancer diagnosis and deliver generalizable, transparent, and precise predictions. Finally, conclusion and future research directions have been presented in section 5.

## 2 Literature review

Among various techniques used for disease diagnosis, DL has emerged as a promising and convincing tool by providing supportive assistance to clinical decisions. Although the advent of Artificial Intelligence (AI) has brought new possibilities, most AI models act like “black boxes” meaning they do not provide information about how a particular decision was reached. This opacity raises concerns about trust and accountability. No medical professional will rely on systems that cannot justify their outputs. In this context, XAI is crucial as it tries to make things clearer and easier to understand. These methods have the ability to bridge the gap by providing enhanced interpretation and explanation of the outcome.

The origins of XAI go back to the 1970s where an expert-system was developed that used a question–answer interface to communicate with users. This was followed by knowledge-based systems in the mid-1980s, and later by the study of artificial neural networks (ANNs) (Volkov and Averkin, 2023). Authors in Volkov and Averkin (2023) highlighted how XAI aimed to address the limitations of DL models being “black box”. It surveyed commonly used XAI techniques and outlined the scope of XAI across radiology, oncology, ophthalmology, and histopathology.

The critical requirement of XAI in clinical settings owing to opaque nature of AI models was also experienced by authors in Dagnaw and El Mouthadi (2023) by claiming that medical experts often express concern about the black-box behavior of such models. To address this challenge, several research directions focused on integrating XAI into medical classification pipelines, such as chest X-ray image analysis for tuberculosis and pneumonia. These studies use lightweight CNNs and Score-CAM visualizations to balance accuracy with interpretability. Interpretable models allow clinicians to audit predictions, validate model behavior, and thus ensure that conclusions are purely based on clinically relevant features. Authors in Latha et al. (2024) present a point-counterpoint discussion on the necessity of interpretable AI in clinical practice and highlighted that DL models may rely on data-source artifacts rather than anatomical features during decision making. Thus, it is well accepted that XAI can be used to audit and verify model behavior.

XAI has been successfully applied across multiple areas of medicine to enhance diagnostic accuracy and interpretability. For instance, Tariq et al. (2024) applied EfficientNet-B7 with Grad-CAM on breast ultrasound images, helping radiologists under-stand the regions associated with malignancy. In Hong and Lin (2024), the authors employed a CNN-based XAI framework for automated skin lesion classification. Talaat et al. (2024) introduced a 3D CNN enhanced with Grad-CAM for breast cancer classification using 3D scans, allowing radiologists to cross-reference model outputs with clinical insights. This approach maintains high accuracy while ensuring interpretability. Similarly, in Tiwari and Agrawal (2024), LIME was applied to predictions from a VGG16 model (98.8% accuracy), offering region-based explanations for clinical understanding. Work in Naz et al. (2023) used Score-CAM with lightweight CNNs to detect pneumonia and tuberculosis in chest X-rays, sup-ported by CLAHE preprocessing. The authors emphasized the practical value of XAI in resource-limited settings. In Karim et al. (2024), DenseNet121 was used for early lung cancer detection, and five XAI techniques were compared. The comparative analysis carried out in Karim et al. (2024) demonstrated that Grad-CAM and Guided Grad-CAM provided the most convincing and clinically relevant explanations. Hoque et al. (2024) further advanced explainable diagnostics by using an ensemble CNN approach in association with Grad-CAM to localize cancerous tissues across multiple histopathology datasets.

In Wedisinghe and Fernando (2024), authors introduced an interpretable geometric approach for plant species identification using Triangle Distance Representation (TDR), where triangle center distance matrix (TCDM) and symbol matrix correspond to specific geometric features of leaves. This underscores the broader relevance of explainability in interpreting shape-based and structural features. These studies collectively demonstrate that XAI methods play an essential role in bridging AI predictions with clinical interpretability across varied applications.

The VGG16 model has been implemented and fine-tuned for predictions in current research owing to its demonstrated competence in a variety of XAI-based medical im-aging. VGG16 is simple, moderately efficient, and provides a good balance between speed and accuracy. For instance, Authors in Jia et al. (2020) used VGG16 with Layer-wise Relevance Propagation (LRP) for disease diagnosis in corn leaf by generating interpretable heatmaps. Authors in Ahmed et al. (2024) also fine-tuned VGG16 for kidney stone detection in Ureter, Kidney, Bladder X-ray (KUB) X-ray images. It’s simple architecture and strong performance established its suitability for tasks that need binary medical classification. An ensemble of VGG16, InceptionV3, and ResNet50 was also experimented by authors in Kumaran et al. (2024) for a three-class lung cancer dataset, where VGG16 contributed significantly to low-level feature extraction. Pallavi et al. (2024) used VGG16 to develop a mobile application for medicinal leaf classification, demonstrating its light-weight and deployable nature.

In Hong and Lin (2024), the authors assessed top-5 ImageNet performance across multiple models, with VGG16 emerging as one of the strongest performers. Ahmed et al. (2023) used a modified VGG16 for binary brain tumor classification, supported by LRP visualizations. Similarly, in Yang and Wei (2019), transfer learning with VGG16 was used for cataract detection, achieving a test accuracy of 96.1%. VGG16 also performs well with augmented data, as shown in Yang et al. (2022), reinforcing and advocating authors’ reliance on VGG16 in current study. As listed, numerous researchers have attempted to employ VGG16 for disease diagnosis and classification. A summary of notable research findings is presented in Table 1.

**Table 1:** Latest research in the domain.

Authors and Year	Model	XAI technique	Application	Key contribution
Dagnaw and El Mouthadi (2023)	Lightweight CNN	Score-CAM	Pneumonia & Tuberculosis (Chest X-Ray)	Combines classification with XAI for transparency and clinical interpretability.
Jia et al. (2020)	DNN (example)	XAI in general	Clinical AI Systems	Emphasizes need for interpretability to avoid bias and enable auditing.
Latha et al. (2024)	EfficientNet-B7	Grad-CAM	Breast Cancer (Ultrasound)	Balances performance with clinical explainability.
Tariq et al. (2024)	VGG16	LRP	Corn Leaf Disease	Best classification accuracy among tested models, highlighted decision regions.
Ahmed et al. (2024)	Modified VGG16	LRP	Kidney Stone (KUB X-Ray)	Effective binary classifier with support from heatmap visualizations.
Yang et al. (2022)	VGG16 vs. ResNet50	Grad-CAM	Pneumonia (Chest X-Ray)	VGG16 outperformed ResNet50 on small datasets, better interpretation.
Ahmed et al. (2023)	Modified VGG16	LRP	Brain Tumour (MRI)	Binary classifier supported by LRP-based interpretability.
Kumaran et al. (2024)	VGG16, ResNet50, InceptionV3 Ensemble	Grad-CAM	Lung Cancer	Ensemble model for better feature learning; supported by visual XAI.
Hong and Lin (2024)	CNN (VGG16)	LIME	Skin Lesion	Local interpretability through LIME improves clinical trust.
Singh et al. (2023)	VGG16 (Transfer Learning)	Not Mentioned	Cataract Eye Disease	High performance via fine-tuning, supports early detection.
Tiwari and Agrawal (2024)	VGG16	LIME	Brain Tumour (MRI)	Visual superpixel explanations improve decision-making clarity.
Pallavi et al. (2024)	VGG16	Not Mentioned	Medicinal Leaf Classification	High accuracy mobile solution using transfer learning.
Karim et al. (2024)	Ensemble: VGG, ResNet, DenseNet, EfficientNet	Grad-CAM	Multi-Cancer (Histopathology)	High accuracy and interpretability using ensemble and Grad-CAM.

Despite the significant advancements and widespread integration of XAI techniques with DL across medical imaging domains, authors have identified few research gaps. First, most studies focus on specific modalities such as chest X-rays, retinal scans, or skin lesions, explainability for histopathological image analysis (for colon cancer) has still taken a backseat. The reason for this may be complex structural and textural variations in histopathological images. Secondly, many existing works rely primarily on qualitative XAI evaluation, lacking quantitative assessment metrics that quantifies explanation validity and consistency. Third, previous studies often focus on a single XAI technique (e.g., only Grad-CAM or only LIME), leaving room for comparative or hybrid approaches that provide a more comprehensive interpretation.

Current research work aims to address these gaps by implementing and compares multiple XAI methods—Grad-CAM, LIME, and SHAP—on colon cancer histopathological images using a fine-tuned VGG16 architecture. Through this approach, authors in current research aim to enhance both classification accuracy and interpretability, ensuring that the decision-making process aligns with clinically relevant visual cues. Having established the background and motivation through the literature review, the following section presents the dataset used in this study, detailing its source, structure, and preprocessing steps.

## 3 Proposed methodology

**Figure 1:**
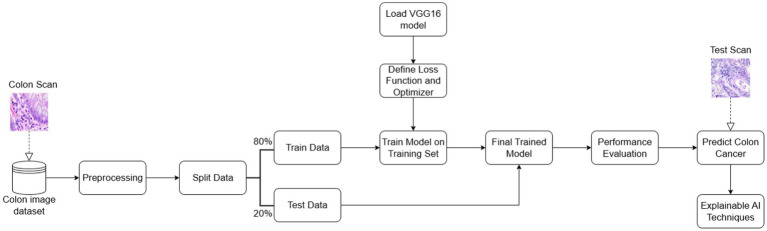
Proposed methodology pipeline.

The proposed solution has five main stages as illustrated in Figure 1. These stages include dataset acquisition and preprocessing, model training, calculation of 2D symmetry indicators, model performance evaluation, and enabling XAI techniques and their comparison.

### 3.1 Dataset acquisition

**Figure 2:**
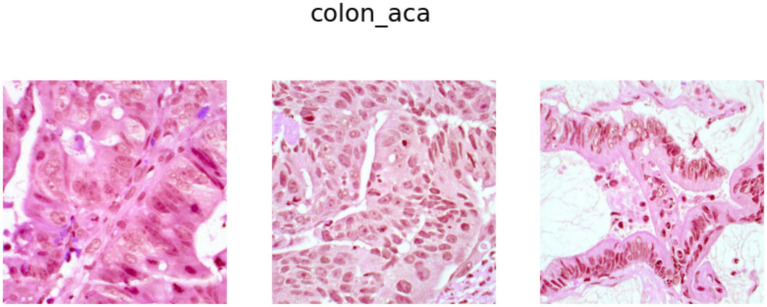
Colon adenocarcinoma samples.

**Figure 3:**
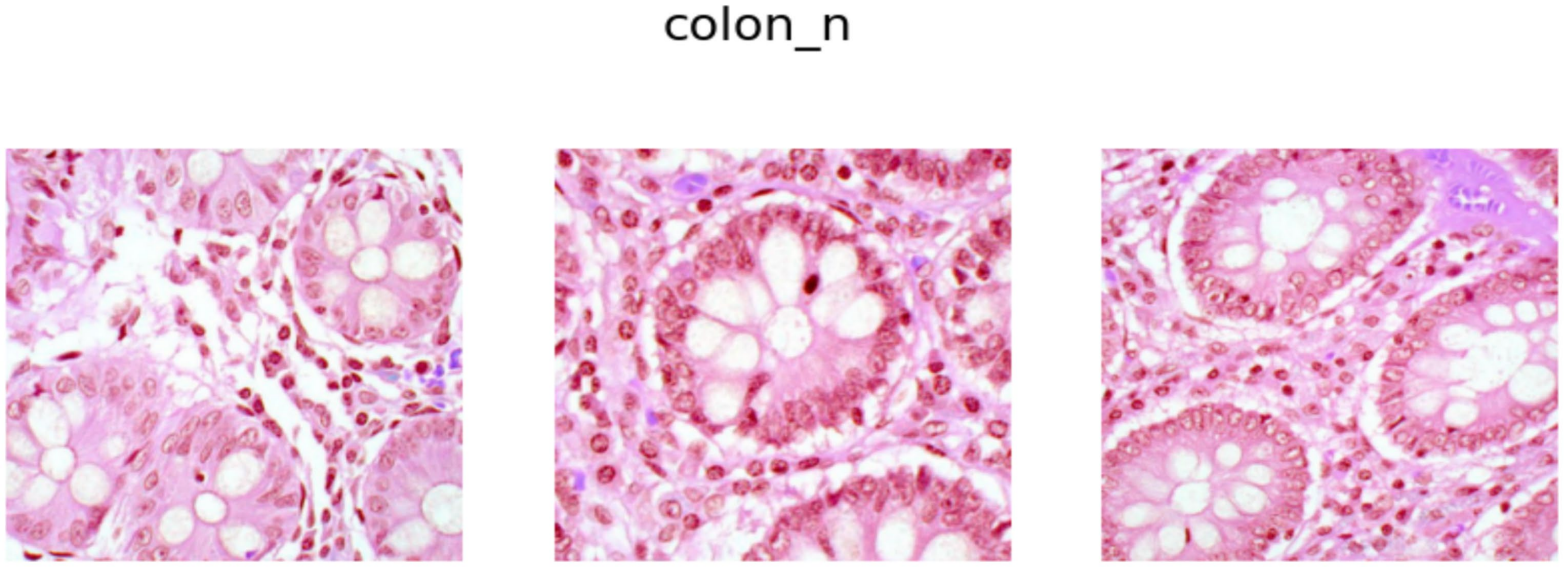
Benign colon tissue samples.

A DL-based binary classification model is trained on a histopathological image dataset consisting of 25,000 labeled images (Borkowski et al., 2019). The considered dataset consists of high-resolution hematoxylin and eosin (H&E) stained images evenly distributed across 5 different classes namely Lung Benign Tissue ( lung_n ), Benign Colon Tissue ( colon_n ), Lung Adenocarcinoma (lung_aca ), Colon Adenocarcinoma ( colon_aca ), and Lung Squamous Cell Carcinoma ( lung_scc ). As the current research is primarily focusing on colon cancer, authors have considered only 10,000 colon cancer images ( colon_n and colon_aca ). Further, it is worth mentioning that 5,000 images for each class have been generated through controlled augmentations on 250 original images. Examples of images from the Colon Adenocarcinoma class and Benign Colon Tissue class are shown in Figures 2, 3, respectively.

### 3.2 Data preprocessing

The images in the considered dataset are H&E stained and therefore stained separation needs to be carried out. For the same, authors initially experimented with stained separation technique namely Macenko method (Sharma A. et al., 2023; Jain et al., 2023) during preprocessing with an intention to enhance model performance. However, employment of stain separation technique surprisingly led to marginal performance decline. The observed performance decline may be attributed to the lack of detailed information regarding the staining processes used during dataset augmentation. Variations in staining protocols—such as differences in dye concentration, lighting conditions, and imaging equipment—are likely to have been present; however, such metadata is not available in the public domain, limiting the effectiveness of stain normalization techniques.

While sophisticated staining normalization techniques could not be leveraged owing to the lack of stain information, authors carried out standard pixel normalization to a uniform range (0, 1) from (0, 255). After that, the dataset is split into data for training and data for testing in ratio of 80:20. Further, 20% of the data from the training set was used for validation purposes.

### 3.3 Model training

The model is developed on top of the VGG16, a standard pre-trained CNN. Based on the evaluation of different models in the above sections, it is apparent that the VGG16 model fine-tuned on the dataset gives the best results. During training, this model learned high-level features from fine-tuning convolutional layers of VGG16, which were useful for classifying colon Adenocarcinoma and benign colon tissue. Also, the fully connected layers of the underlying VGG16 are replaced by a new classifier for binary classification. The architecture is wrapped using the Sequential API to enable simple integration. The final layer comprises of a single neuron for binary classification. Parameters used during model training are mentioned in Table 2.

**Table 2:** Parameters values for VGG 16 model training.

Parameters	Value
Optimizer	Adam
Loss function	Binary Cross-Entropy
Learning rate	0.0001
Epochs	10
Batch size	32

### 3.4 Calculation of 2D symmetry indicators

In colon histology, as discussed earlier, symmetry is not only an aesthetic characteristic but also a biological marker of tissue integrity. Healthy colon tissue shows (1) radial symmetry in round glandular structures; (2) bilateral symmetry in epithelial layers, and (3) repeated symmetrical patterns in mucosal glands. Colon Adenocarcinoma disturbs these patterns because of alterations in cellular architecture. To formalize this approach, authors evaluate several 2D symmetry indicators for both benign and malignant samples. These symmetry cues enhance the proposed DL model by supplying clinically interpretable indicators that correlate with malignancy. Adding symmetry-based observations directly supports the goals of this Special Issue by showing how geometric and structural symmetry helps us make sense of visual data in medical imaging.

### 3.5 Performance evaluation

During performance evaluation, various performance metrics are considered to determine the effectiveness of the proposed method. Some of the considered performance metrics are as follows:

#### 3.5.1 Training and validation performance

**Figure 4:**
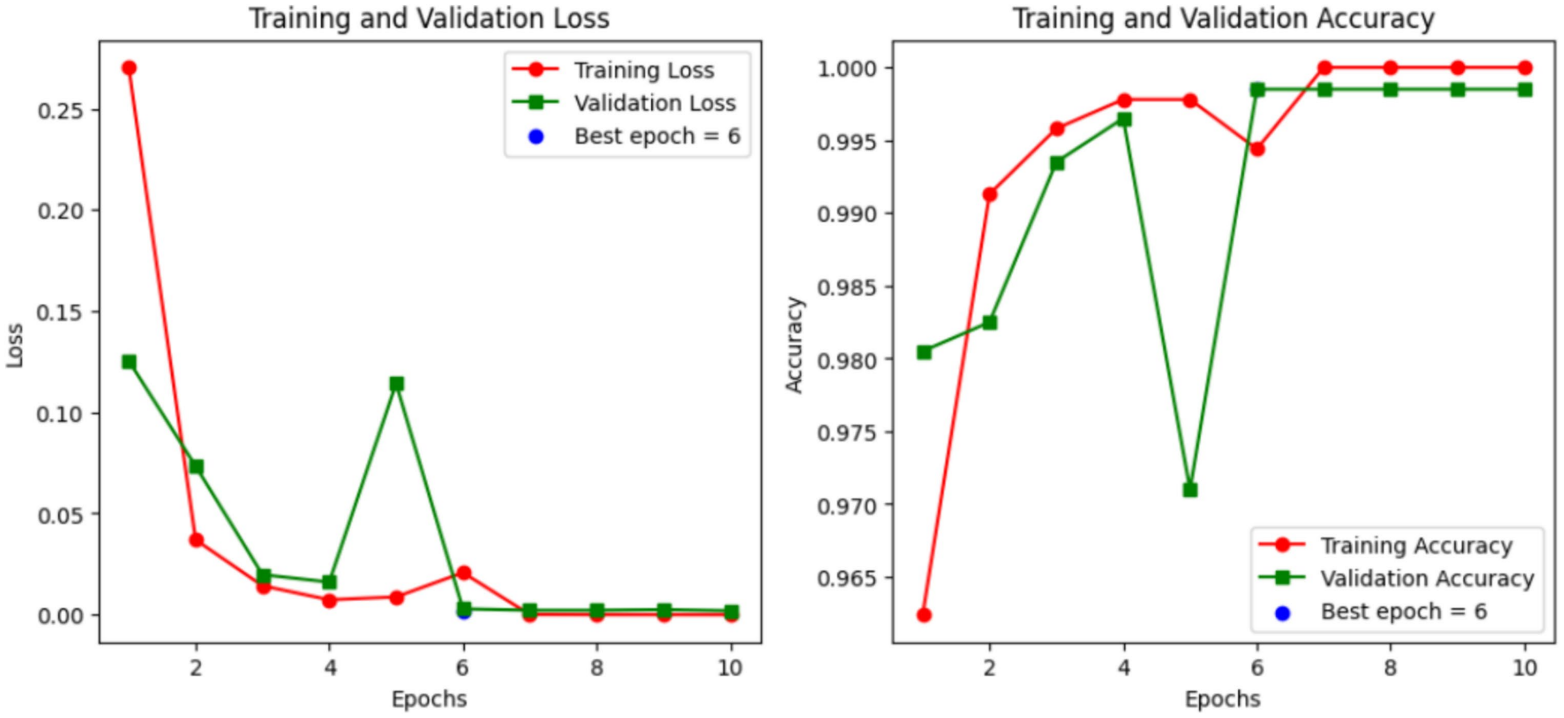
Training and validation curves.

The loss curve (during training & validation) and accuracy curve during successive epochs is pictorially illustrated in Figure 4. Figure 4 clearly represents that accuracy and loss both becomes stagnant after few epochs (epoch 6 in current scenario), demonstrating the steep convergence.

#### 3.5.2 Analysis of confusion matrix

**Figure 5:**
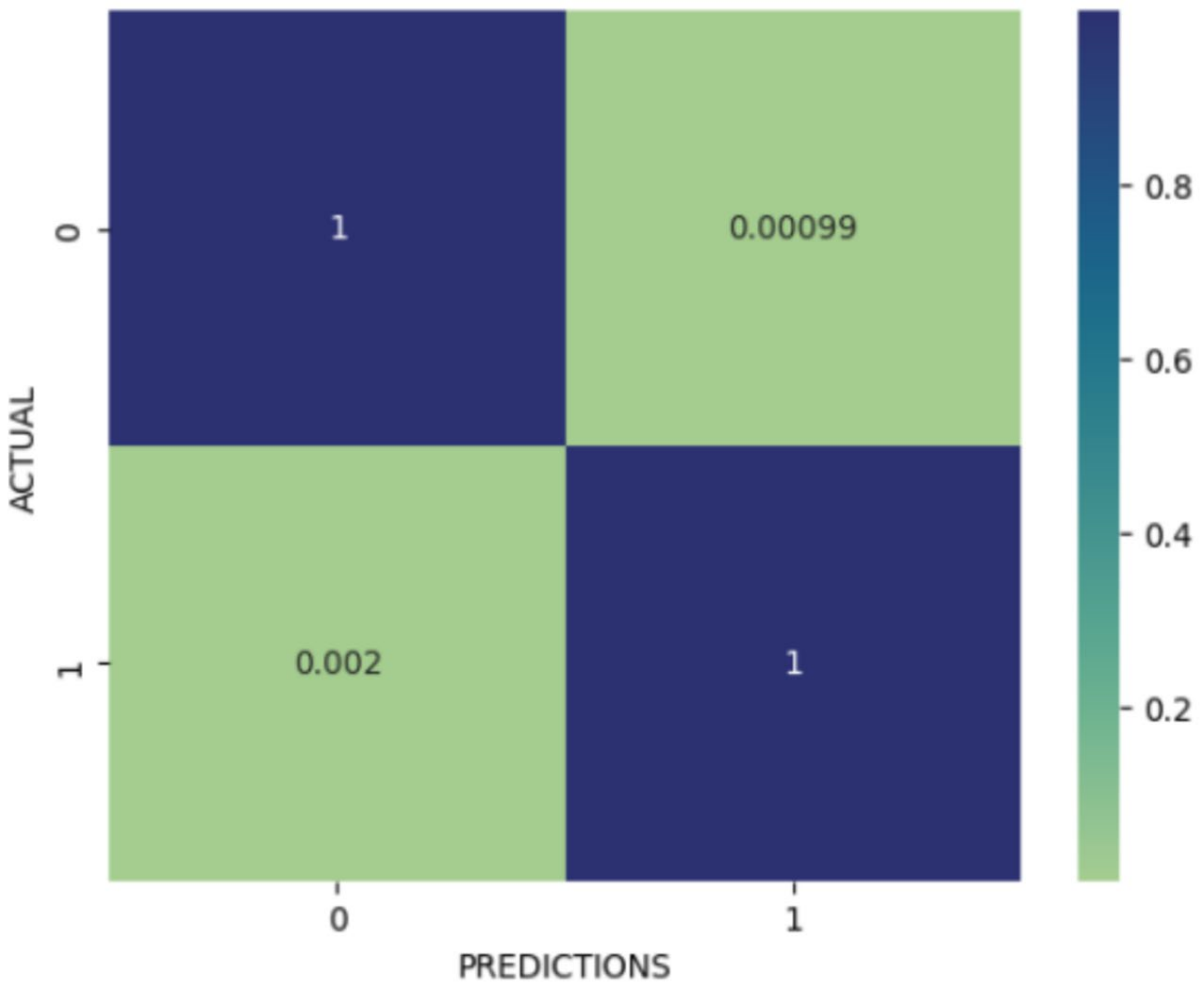
Visualization of confusion matrix.

A complete assessment of the binary classification is illustrated in Figure 5 through confusion matrix. With values close to 1, the correctly classified instances are represented by the diagonal elements and indicate almost perfect classification for both classes (Mangla et al., 2022). Confusion matrix clearly indicates that misclassification rate is quite minimal.

The experimentation achieved an accuracy of 99.85% during testing (Singh et al., 2023; Xu et al., 2025) validating efficiency of the model for colon cancer image classification. The low misclassification rate is reflected by a loss value of 0.00079575.

#### 3.5.3 Metrics for classification

The model’s strong performance is also validated by the classification metrics namely precision, recall, and F1-score as illustrated in Table 3. Considered metrics indicate that the model is highly consistent toward detection of colon cancer as it achieves promising recall and precision.

**Table 3:** Classification metrics.

Metric	Class 0 (Normal)	Class 1 (Cancerous)	Macro Avg	Macro Avg
Precision	0.998	0.999	0.9985	0.9985
Recall	0.999	0.998	0.9985	0.9985
F1-score	0.9985	0.9985	0.9985	0.9985

### 3.6 XAI techniques

For the enhanced interpretation of the model predictions, three XAI techniques namely LIME, SHAP, and Grad-CAM are integrated in the current study. This enables achieving visual and numerical interpretations of the model’s outcome.

A LIME

**Figure 6:**
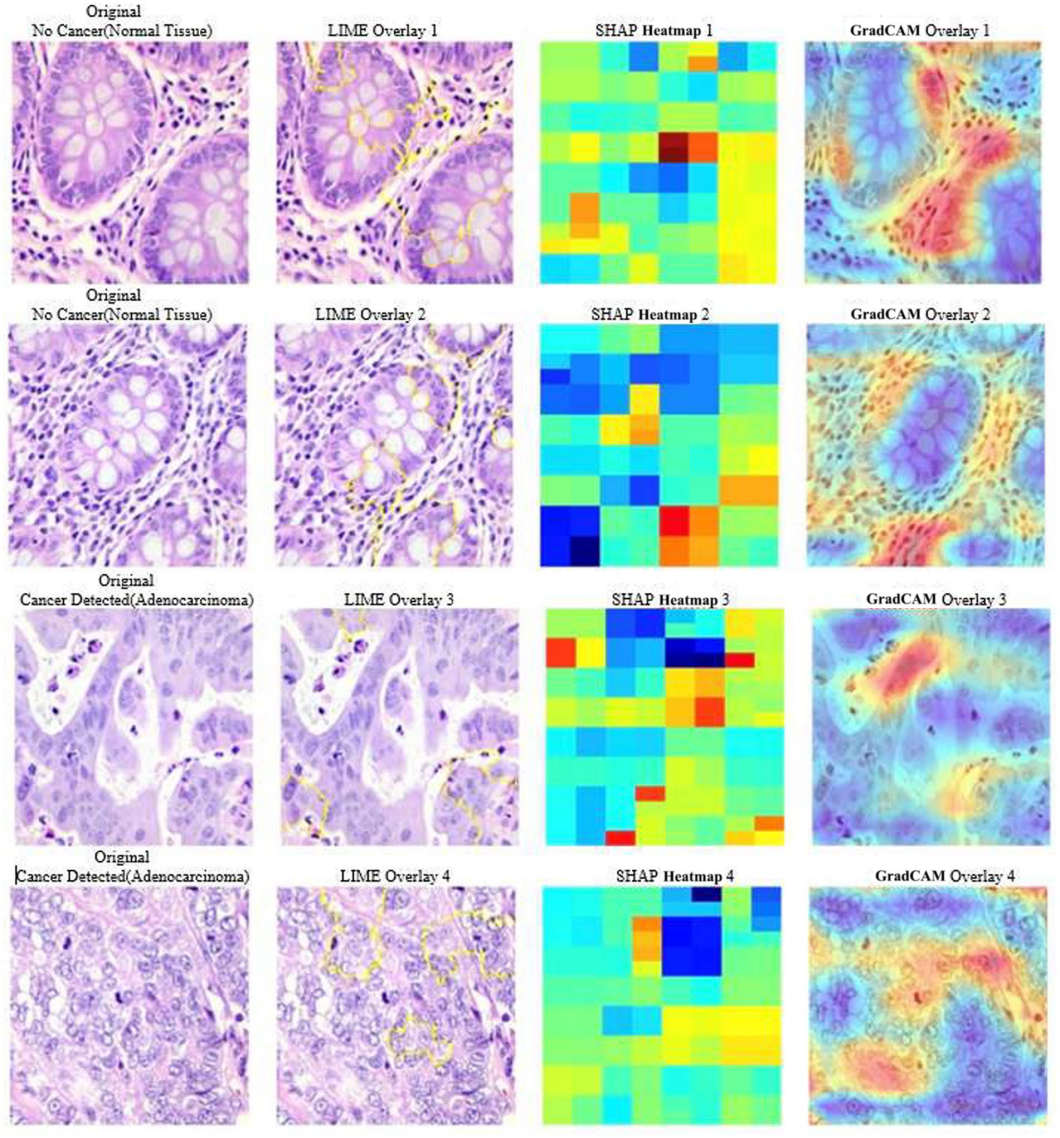
XAI visualizations (Grad-CAM, LIME, SHAP).

LIME is a XAI technique that explains individual predictions by ablation of input images and investigation of the model’s response. This yields a method of highlighting image regions that are the most relevant to a classification decision. For current visual results, LIME applies yellow-highlighted portions on top of the original histopathology images, as presented in Figure 6. Highlighted portions represent portions of the image that significantly contributed toward model’s classification choice. It highlights the superpixels whose removal or modification yields maximum local effect on model prediction. Thus, LIME mainly focuses on distinctive cellular structures and morphological patterns that differentiates colon_aca tissue from colon_n tissue.

B SHAP

SHAP is a game-theory-based technique that searches for feature contrasts within image area to allow contributions toward the prediction, to achieve higher interpretability at pixel level. SHAP heatmaps illustrate positive and negative regions that contribute to the prediction of a class. It assigns a Shapley value to every pixel or region based on the contribution it makes in pushing a model toward a particular class, as shown in Figure 6. SHAP’s results confirm biological validity in our model predictions since some differentiating cellular patterns had significant impact on classification.

C Grad-CAM

Grad-CAM supports the process of creating visual explanations by assigning salient image locations significantly contributing toward model’s prediction. Although LIME and SHAP give pixel-level information, Grad-CAM provides a larger activation map superimposed on the input image, as shown in the last column of Figure 6. The results illustrated in Figure 6 indicate that the DL model considers major structural parts of the tissue for benign and Colon Adenocarcinoma classification. In addition, the regions demarcated in red show that the model correctly identifies key pathological features that are consistent with colon cancer.

D Symmetry-Aware Intelligence from XAI Findings

The XAI visualizations enable to identify the regions where natural glandular symmetry is disrupted. Specifically for benign tissue, Grad-CAM and SHAP contribute toward highlighting circular, well-organized, repetitive symmetric gland structures. On the other hand, XAI methods emphasize irregular, asymmetric regions, fragmented epithelial boundaries, and loss of radial symmetry for Colon Adenocarcinoma. LIME’s superpixel segmentation are also in agreement with the observation that benign superpixels tend to form coherent, symmetric clusters, whereas cancer patches yield irregular and highly asymmetric superpixel shapes.

By linking XAI outputs with symmetry patterns, authors demonstrate that the model identifies biologically meaningful symmetry-breaking cues. This symmetry-aware interpretation strengthens the clinical reliability of proposed framework and directly aligns with the symmetry theme. Additionally, application of XAI models enhance the belief and trust in model’s outcome as it learns the appropriate histological features and not only image acquisition artifacts. During comparative analysis of different interpretations, it is observed that LIME, SHAP, and Grad-CAM outputs are highly coherent with each other, thus appear to be stable and consistent, establishing a strong and reliable model.

## 4 Results and discussion

As discussed, the proposed methodology is experimented on the publicly available dataset comprising of 25,000 images. Out of these 25,000 images, current experiment focused on only 10,000 images. Our model performed substantially well in classification. The obtained loss is quite low and accuracy is achieved to be 99.85%. As mentioned previously, the confusion matrix analysis also witnessed that the model is nearly flawless in classifying things. There were not many false positives or false negatives. The categorization report also confirmed that the normal and malignant classifications had very good precision, recall, and F1-scores.

Authors used various XAI models to explain the model’s decision-making process and presented a comparison among them. Here, LIME presents the most important superpixels, pointing out regions which, if changed, would cause a major shift in the model’s prediction. This helps in comprehending which regional features contribute more toward a particular classification result. On the other hand, SHAP assigns importance scores to different image regions, locating cancerous (red) and non-cancerous (blue) areas of an image. This provides insight into how different regions contribute to classification from a global perspective. Grad-CAM has generated coarser activation maps that highlight structural regions of interest in an image that the model places more emphasis on during classification. Of these XAI models, Grad-CAM is the most computationally efficient and best suited for visualization of deep CNN models, whereas LIME is most efficient and best suited for providing local explanations. Further, SHAP model, although slower, provides the most sophisticated global perceptions.

Now, as the current method integrates these three XAI techniques, there is a stronger and more reliable explanation. Convergence of LIME, SHAP, and Grad-CAM all converge on overlapping areas escalates the reliability of the model’s prediction, indicating that these regions are indeed crucial for classification. The agreement of the methods guarantees that the model will not only be correct but also explainable and reliable for clinical decision-making. Therefore, although Grad-CAM is faster and scalable, having the trio of SHAP, LIME, and Grad-CAM enables the proposed method to achieve greatest degree of confidence in the model’s decision process, creating a robust and explainable classification.

### 4.1 Evaluation of models

Authors evaluated four different CNNs, namely ResNet50, EfficientNetB0, DenseNet121, and VGG16. Here, each model was trained using the same hyperparameters to ensure fairness. The performance metrics obtained through various models are illustrated in Table 4. It is evident from Table 4 that DenseNet121 and VGG16 achieves high performance, but VGG16 emerges as the best-fitted model as it outperforms the other one. Its accuracy is in close alignment to precision, recall, and F1-score, suggesting a high degree of specificity and sensitivity.

**Table 4:** Performance evaluation summary of various ML models.

Model	Accuracy	Precision	Recall	F1-Score
EfficientNetB0	0.5000	0.250000	0.5000	0.333333
ResNet50	0.7520	0.752004	0.7520	0.751999
DenseNet121	0.9900	0.990031	0.9900	0.990000
VGG16	0.9985	0.998462	0.9985	0.998800

### 4.2 Cross validation analysis

In order to enhance the statistical power of experiment, authors performed K-fold cross-validation. A 5-fold stratified setup was implemented on the dataset of 10,000 histopathology images in order to keep the balance among classes in each fold. During each run, dataset is divided into training, validation, and testing in the ratio of 80:10:10 and rotated through five folds. All experiments were carried out independently for VGG16, ResNet50, EfficientNet-B0, and DenseNet121 under the same settings. Each of the models processed input images of 224 × 224 pixels, in a 32 batch size, and trained for 10 epochs for every fold. Also, class weights were computed dynamically for every fold to address class imbalance. The classification head also featured a dropout layer with a dropout rate of 0.4 or 40% to reduce overfitting. These hyperparameters and configurations for training guarantee a fair and consistent comparison of model performances, which has been summarized in Table 5.

**Table 5:** Performance evaluation summary of various models.

Model	Avg accuracy	Avg precision	Avg recall	Avg F1 score
DenseNet121	0.988488	0.250000	0.5000	0.333333
ResNet50	0.732274	0.752004	0.7520	0.751999
VGG16	0.992436	0.990031	0.9900	0.990000
EfficientNetB0	0.560294	0.998462	0.9985	0.998800

All five folds demonstrated consistently good performance in cross-validation for each of the models to be evaluated. All models yielded stable learning and low variance, indicating that the models were not overfitted. In these tested CNNs, DenseNet121 and VGG16 appear to outperform other models in terms of precision, recall, F1-score, and accuracy. Overall stability found during K-fold cross-validation adds more evidence to the reliability and robustness of DL models for colon cancer image classification and further supports their applicability in real clinical settings.

### 4.3 Statistical significance and variability analysis

Authors have also performed statistical analysis in terms of mean accuracy, standard deviation, and 95% confidence interval across different runs to compare the performance of CNN models on colon histopathology images. The accuracy and F1-score of considered CNN models are illustrated in Table 6 while mean, standard deviation, and confidence interval score are given in Table 7.

**Table 6:** Accuracy summary.

Model	Mean	Std	95% CI
DenseNet121	0.988488	0.000893	0.001109
EfficientNetB0	0.560294	0.000719	0.000893
ResNet50	0.732274	0.001255	0.001558
VGG16	0.992436	0.001361	0.001690

**Table 7:** F1 score summary.

Model	Mean	Std	95% CI
DenseNet121	0.988734	0.001746	0.002169
EfficientNetB0	0.560216	0.002302	0.002858
ResNet50	0.727194	0.002002	0.002486
VGG16	0.992594	0.001098	0.001363

DenseNet121 and VGG16 models uniformly outperforms the rest by yielding mean accuracy rates of 98.84% and 99.24%, respectively. Additionally, these models achieve high F1-scores indicating that models have high precision as well as sound recall toward classification of cancerous tissue classes. Low standard deviations and narrow confidence intervals clearly indicates that these models have little variance across different runs, indicating sound and consistent predictive behavior. On the contrary, EfficientNetB0 performs miserably by achieving a mean accuracy of 56.02% and F1-score of 56.02%, reflecting its lower ability to learn discriminative features. ResNet50 offers mid-level performance by yielding mean accuracy and F1-score around 73%. The results are consistent across all models underlining the strength of proposed evaluation methodology. It is worth mentioning that outperformance of VGG16 and DenseNet121 is reflective of prior studies indicating their robustness when fine-grained feature representation becomes essential. Therefore, VGG16 and DenseNet121 are good candidates for further development with XAI techniques to produce clinically trustworthy diagnostic systems.

To compare VGG16 and DenseNet121 through statistical evidence, a paired t-test is carried out as the models are processed on the same data folds. The results of the paired t-test for comparison of different models are given in Table 8. The paired t-test between DenseNet121 and ResNet50 yields a *t*-value of 519.6429 with a *p*-value less than 0.0001, indicating that the difference is highly significant. During paired *t*-test of DenseNet121 with EfficientNetB0, t-value is 689.0812 with a *p*-value less than 0.0001 again confirming that there is a significant difference in the performance of these models, with DenseNet121 outperforming EfficientNetB0. The paired *t*-test of DenseNet121 and VGG16 achieves a *t*-value of 6.8266 with *p*-value of 0.0024 suggesting slightly better performance of VGG16 in comparison to DenseNet121 on this dataset. Although exceptional performance was demonstrated by both models, VGG16 appeared to be marginally superior. Lastly, *t*-test for VGG16 versus EfficientNetB0 achieves a dramatic result by obtaining a *t*-value of 501.1394 and a p-value of less than 0.0001, indicating a substantial difference in performance.

**Table 8:** Paired *t*-test summary.

Comparison	*t*-value	*p*-value	Statistically significant
DenseNet121 vs ResNet50	519.6429	0.0000	Yes
DenseNet121 vs EfficientNetB0	689.0812	0.0000	Yes
VGG16 vs DenseNet121	6.8266	0.0024	Yes
VGG16 vs EfficientNetB0	501.1394	0.0000	Yes
ResNet50 vs EfficientNetB0	200.1229	0.0000	Yes
ResNet50 vs VGG16	-475.8030	0.0000	Yes

Authors also compared EfficientNetB0 with ResNet50 using a paired *t*-test. This test gave a *t*-value of 200.1229 and a *p*-value of less than 0.0001, which shows that there is a statistically significant difference in favor of ResNet50. Although ResNet50 performed moderately in general, it still beats EfficientNetB0. Lastly, the paired *t*-test between VGG16 and ResNet50 returned a *t*-value of −475.8030 with a *p*-value less than 0.0001, showing a clear indication of significance. The VGG16 model significantly outperformed ResNet50, further justifying its suitability for high-precision colon histopathology classification. Resultantly, these paired *t*-tests categorically establish that highest performance is achieved by VGG16 in comparison to other models.

### 4.4 Symmetry quantification experiment

To validate the link between tissue malignancy and structural symmetry, authors conducted a lightweight analysis using three classical symmetry metrics as follows:

- Reflection Symmetry Score (RSS): Measures similarity between left–right or top-bottom halves of the image region.
- Radial Symmetry Transform (RST): Quantifies circular or radial uniformity around a central point.
- Symmetry Index (TSI): Computes self-similarity patterns using Local Binary Patterns (LBP).

The results of these symmetry quantification analysis are illustrated in Table 9. Here, it is obvious from the obtained results that benign tissues preserve symmetric glandular structures while cancerous tissues show substantial breakdown of these patterns, establishing symmetry as an auxiliary biomarker to aid DL model’s decision-making process. It also helps demonstrate that the XAI visualizations align with symmetry-based abnormalities.

**Table 9:** Symmetry analysis of both classes.

Class type	RSS	RST	TSI
Class 0 (Normal)	0.71	High radial peaks	High self similarity
Class 1 (Cancerous)	0.36	Weak radial patterns	Low self similarity

### 4.5 External validation and variability analysis

In order to test and validate the generalizability of proposed model, authors performed external validation on various other histopathological image datasets. These datasets include images for colorectal cancer, lung cancer, gastrointestinal tract cancer, and gastric cancer. Following is the description of the four external datasets:

- Lung cancer histopathology dataset: This dataset consists of three classes namely lung benign tissue, lung squamous cell carcinoma, and lung adenocarcinoma. Of these, authors utilized only lung benign tissue and lung adenocarcinoma. Each class consisted of 5,000 images. Since lung adenocarcinoma exhibits a morphology similar to colon, this dataset is significant for external validation.
- Colorectal cancer histology dataset: This dataset consists of a total of 5,000 images distributed evenly across eight classes – Simple stroma, Tumour epithelium, Complex stroma, Debris and mucus, Immune cells, Mucosal glands, Adipose tissue, and Background (Mader, 2018). From these classes, Tumour epithelium (similar to Colon Adenocarcinoma) is used to check for cancerous tissues, and Simple stroma and Mucosal glands are treated as benign tissues. The rest of the classes show a different formation of cancerous, benign, or a mixture of both which is absent in colon. Hence, they were not considered for external validation and hence only 1875 images out of 5,000 images were used for validation.
- Gastric cancer dataset: The gastric dataset contained eight histological classes organized in eight folders (Orvile, 2021). For the purpose of external validation, only the TUM (adenocarcinoma) and NOR (normal mucosa) classes were used.
- Gastrointestinal (GI) tract cancer dataset: This is a comprehensive GI endoscopic image dataset (Pogorelov et al., 2017). It has eight classes belonging to benign and cancerous categories. Here, authors selected only three classes namely normal-pylorus, normal-cecum, and normal-z-line as these classes are indicative of benign structures. The classes namely polyps, dyed-lifted-polyps, ulcerative-colitis, and esophagitis are used for cancer prediction while dyed-lifted-polyps class is not used for external validation.

The considered datasets share key features like mucin production, gland formation, and nuclear atypia similar to Colon Adenocarcinoma tissue (Mayo Clinic Staff, 2025; Zhang et al., 2019). Hence, authors tested current model to establish its ability to differentiate between Colon Adenocarcinoma tissues and benign tissues found in these four regions. Obtained results have been summarized in Table 10. The results shown in Table 10 clearly establishes the model’s capability in differentiating Colon Adenocarcinoma tissues from benign tissues. This can be extremely useful in clinical settings of preliminary scanning of cancer detection. It also proves that models trained on Colon Adenocarcinoma have an ability to detect morphologies of adenocarcinoma tissues.

**Table 10:** Analysis for generalization.

Metric	Mean	Std Dev	95% CI
Lung cancer			
Accuracy	0.9933	0.0020	0.9915–0.9951
Precision	0.9986	0.0012	0.9976–0.9996
Recall	0.9880	0.0039	0.9846–0.9914
F1-score	0.9933	0.0020	0.9915–0.9950
Colorectal cancer			
Accuracy	0.9024	0.0038	0.8991–0.9057
Precision	0.8551	0.0082	0.8480–0.8622
Recall	0.9104	0.0115	0.9003–0.9205
F1-score	0.8818	0.0047	0.8777–0.8860
Gastric cancer			
Accuracy	0.7965	0.0132	0.7850–0.8081
Precision	0.6532	0.0188	0.6367–0.6697
Recall	0.8323	0.0204	0.8144–0.8501
F1-score	0.7318	0.0153	0.7184–0.7451
Gastrointestinal cancer			
Accuracy	0.4178	0.0124	0.4069–0.4286
Precision	0.8358	0.0636	0.7801–0.8915
Recall	0.0844	0.0163	0.0701–0.0987
F1-score	0.1531	0.0280	0.1285–0.1777

In order to test the generalizability of proposed VGG16-based model, which is trained on colon cancer, authors further validate it on multiple external cancer datasets based on a 5-fold stratified cross-validation setup. Each fold is trained for 10 epochs with a batch size of 32 images of 224 × 224 pixels. The classification head consists of a dropout layer with a rate of 0.4 to reduce overfitting and improve model robustness. During training each of these datasets underwent class weighting and data augmentation-including random flips, brightness, and contrast adjustments-were used in order to deal with class imbalance and improve generalization. This allowed authors to assess the performance of our model on unseen data across different cancer types. It has shown notable performance for lung cancer, high reliability for colon cancer, and a generally moderate performance for gastric cancer (especially in precision), and poor performance on broader gastrointestinal cancers, with low recall. These findings underpin aspects where the model performs well on cancers that are within its training data and indicate further areas where improvement will enhance generalization.

A consistent performance across diverse datasets is a preliminary indication of the model’s generalization ability. However, proposed model cannot be claimed as a comprehensive generalized model in its current form but it has potential to be taken forward in the direction of establishing generalizability. The validation regarding generalizability can be further strengthened should there be diverse and sufficient amount of dataset available.

Further, the external evaluation datasets are representative of a wide variety of histologic classifications but may not necessarily reflect the complexity, variability, and uncertainty present in a practical clinical setting. This necessitates further exploration and investigation ahead of its integration into standardized diagnostic modalities.

In all, this external evaluation confirms that VGG16 model, trained for colon cancer, yields strong performance on datasets from similar histopathological tasks but shows somewhat reduced effectiveness as the cancer tissue morphology gradually moves away from that within the training domain. This result points to domain-specific learning and further indicates that more diverse multicancer data or better transfer learning can lead to improvements in cross-cancer generalization.

### 4.6 Ablation studies on XAI techniques

In order to add depth to proposed colon cancer classification model’s interpretability, authors performed ablation studies using several XAI techniques together—Grad-CAM, SHAP, and LIME (Hong and Lin, 2024; Naz et al., 2023; Yang et al., 2022). Although the individual contribution of each method was discussed previously, this section explores the synergy obtained by using them together in pairs: Grad-CAM+LIME, LIME+SHAP, and SHAP+Grad-CAM. These combinations were applied by superimposing the respective visual explanations over the original histopathological image through weighted blending approaches. For example, Grad-CAM heatmaps were created through class activation mappings from the last convolutional layer of the VGG16 backbone, whereas LIME and SHAP explanations added region-based and perturbation-informed relevance overlays, respectively.

**Figure 7:**
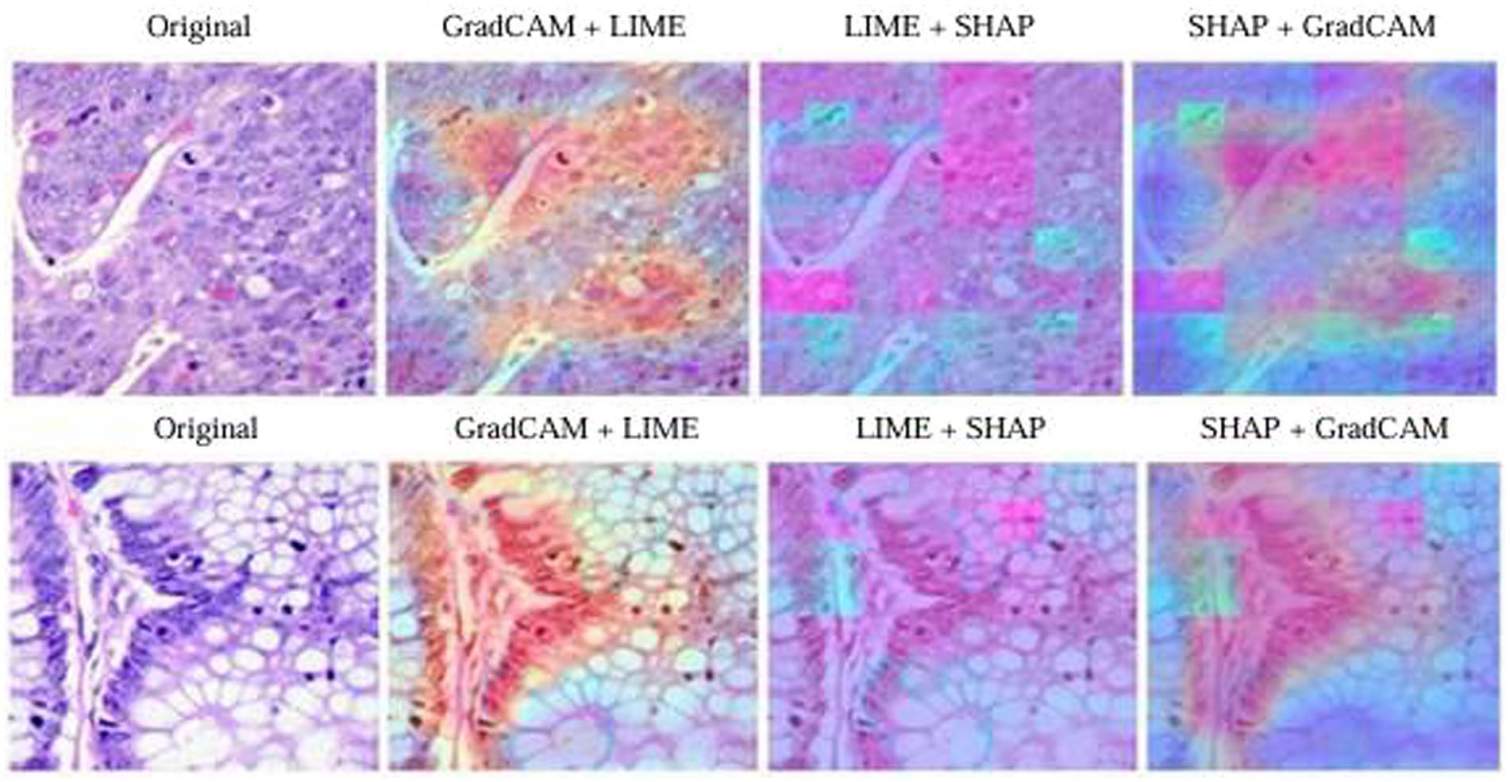
Ablation study results.

The ablation results are illustrated in Figure 7 for a normal tissue prediction clearly demonstrate interpretability enhancement in each combination. When used in isolation, Grad-CAM yielded fast but relatively imprecise localization of salient areas. Similarly, LIME added localized boundary-centered areas while SHAP captured global contextual relationships via pixel perturbation when implemented in isolation. However, when Grad-CAM is integrated with LIME, it gives more focused attention to histological borders. Similarly, LIME in association with SHAP emphasized semantically important glandular areas with higher granularity; and SHAP + Grad-CAM provided a well-balanced global-to-local explanation. Critically, integration of all the methods was the best way to obtain the most extensive and visually consistent interpretation. Such combinations enhanced visual trust by affirming the model’s decision consistency across different XAI paradigms.

To quantitatively assess the accuracy of interpretability, authors evaluated deletion AUC values for each method and their combinations. These values measure how well the highlighted regions map to the relevant areas influencing model predictions. Smaller value of AUC indicates better interpretability. In current experimentation, method blending consistently achieved lower AUCs when compared with individual methods, thus advocating the merits of combined methods in describing model-important features more accurately. These results have been summarized in Table 11.

**Table 11:** Analysis for interpretability using XAI techniques.

Image	SHAP deletion AUC	LIME deletion AUC	Grad−CAM Deletion AUC	SHAP+LIME AUC	SHAP+Grad−CAM AUC	LIME+Grad−CAM AUC	All combined AUC
Image 1	0.6006	0.3614	0.5717	0.3621	0.5717	0.3441	0.3441
Image 2	0.5784	0.2041	0.4029	0.2045	0.4029	0.2681	0.2681

The experimentation of proposed model achieves motivating and convincing results for classifying benign tissue from adenocarcinoma. While the evaluated performance metrics demonstrate encouraging results, the authors acknowledge the potential for misclassification, particularly in rare and ambiguous cases. For rare cases, model may possibly assign an incorrect label, rather than explicitly flagging uncertainty. This limitation of proposed model pertaining to possible incorrect label assignment for rare and ambiguous cases can be primarily attributed to the limited size and diversity of the dataset. This limitation can be addressed by model training on diverse tissues with an aim to achieve performance enhancement toward handling rare and ambiguous cases. Incorporation of confidence thresholding or uncertainty quantification methods (such as Monte Carlo Dropout) may be considered as a future extension to flag low-confidence predictions. Additionally, the study does not assert the identification of any tissue types other than adenocarcinoma and benign tissues.

To address these limitations of possible misclassification of ambiguous cases and handling of only adenocarcinoma and benign tissues, authors would be amenable to carry out experiment on dataset consisting of diverse tissues. Finally, the authors clarify that the proposed system is not intended to serve as a standalone substitute for expert clinical judgment, but rather as a triage-support tool designed to assist pathologists in the diagnostic process.

## 5 Conclusion

Current research presents a highly precise model for the diagnosis of colon cancer using a VGG16 CNN that achieves an encouraging test accuracy of 99.85%. The model exhibited very high precision, recall, and F1-scores for both classes, normal and cancer, as demonstrated by the classification report. With an aim to escalate beyond prediction, authors integrated three high-quality XAI techniques namely LIME, SHAP, and Grad-CAM. These XAI techniques leverages to gain insights regarding classification outcome. Among various XAI techniques, Grad-CAM demonstrated speed and scalability making it an appropriate choice for its large-scale deployment in healthcare. SHAP, though computationally costly, offered theoretical robustness and great insight. LIME was handy in local interpretability, especially convenient in debugging individual predictions. Notably, the region of intersection identified by all three approaches are strong indicators of the medical relevance and reliability of current model. Authors also carried out cross validation to test the effectiveness of proposed model on other cancer types. This synergy of DL and XAI has shown immense scope in computer-aided colon cancer diagnosis. This method can further be deployed in real-world clinical practices after testing it on various other real-world examples and getting it evaluated by clinical experts.

Although the results yielded by current experiment are encouraging, it still has some limitations in terms of possible misclassification of ambiguous cases. Also, the proposed work has been tested only for identifying adenocarcinoma and benign tissues, its performance for other tissues cannot be predicted. In order to address these limitations, current work may be extended in the direction of performance evaluation on more diverse datasets with a much greater variety of colon pathologies, including rare or ambiguous cases. Also, uncertainty estimation models may be integrated to allow the model to identify low confidence predictions. All low-confidence predictions can be passed on to trained pathologists for review and analysis with the goal that this model functions merely as a decision support system and not as a stand-alone diagnostic model.

In summary, although the proposed model produced results that are promising, additional validation of the model in multiple and diverse clinical datasets is necessary prior to the model being used in an actual diagnostic environment.

## Data Availability

The original contributions presented in the study are included in the article/supplementary material, further inquiries can be directed to the corresponding authors.
